# The influence of the vaginal ecosystem on vaginitis, bacterial vaginosis, and sexually transmitted diseases: an epidemiological study and literature review

**DOI:** 10.1007/s00404-024-07626-8

**Published:** 2024-07-11

**Authors:** Sara Occhipinti, Giosuè Giordano Incognito, Marco Palumbo

**Affiliations:** Department of General Surgery and Medical Surgical Specialties, University of Catania, Via Santa Sofia 78, 95123 Catania, Italy

**Keywords:** Vaginal microbiota, Bacterial vaginosis, Papilloma virus, *Chlamydia trachomatis*

## Abstract

**Purpose:**

This study aimed to demonstrate the correlation between altered balance of the vaginal ecosystem and increased risk of vaginitis, bacterial vaginosis, and sexually transmitted diseases and the association between specific alterations found in fresh bacterioscopic examinations (FBE) and the risk of certain infections.

**Methods:**

A retrospective, monocentric study was conducted from January 2013 to December 2023. Patients who underwent FBE and vaginal swabs following reported symptoms or suspected syndromic pictures of vulvovaginal infections were included.

**Results:**

Two thousand one hundred ten patients were included and divided into a control group (*n* = 811, 38.4%) and a pathological group (*n* = 1299 patients, 61.6%), based on the presence of alterations at the FBE. In the pathological group, 1185 women (91% of positive FBE) had vaginal infections detected through vaginal swabs. The presence of lactobacilli and typical inflammatory cells was detected in 111 (8%) women with pathological FBE and correlated with higher rates of positive swabs for common germs (*n* = 104, 94%), often leading to co-infections (*n* = 30, 29%). Conversely, Döderlein’s cytolysis (*n* = 56, 4.3% of positive FBE) indicated a predominance of positive human papillomavirus (HPV) swabs (*n* = 33, 59%). The presence of fungal elements (*n* = 208, 16% of positive FBE) suggested a higher prevalence of co-infections (*n* = 62, 30%). Similarly, mixed bacterial flora (*n* = 470, 36% of positive FBE) and *Trichomonas vaginalis* (*n* = 11, 0.8% of positive FBE) correlated with positive swabs for other pathogens, except for Mycoplasma (*n* = 0). Bacterial vaginosis (*n* = 443, 34% of positive FBE) was linked to co-infections (*n* = 142, 32%) and HPV (*n* = 123, 28%).

**Conclusion:**

The importance of conducting FBE in patients with vulvovaginal symptoms is emphasized. This approach aids in determining the need for further diagnostic tests like vaginal swabs, guided by microscopic findings. A strong correlation emerges between the presence of specific alterations in the FBE and an increased prevalence of certain infections.

## What does this study add to the clinical work?

**Table:** 

A strong correlation emerges between the presence of specific alterations in the fresh bacterioscopic examination and an increased prevalence of certain infections.	
These findings may potentially influence clinical management and therapeutic approaches in these patients.	

## Introduction

The vagina is considered a continuously balanced ecosystem where endogenous and exogenous factors play a significant role in lower tract pathology [1]. Vaginal secretions consist of a combination of substances derived from vulvar secretions from the Bartholin’s and Skene’s glands, shed epithelial and cervical cells, cervical mucus, fluids from endometrial and tubal sources, microorganisms along with the products of their metabolism [2]. The vaginal microbiota of healthy women consists of a variety of aerobic and anaerobic microorganisms in perfect balance, a condition called ‘eubiosis’ [3]. Lactobacilli are the most widely represented subpopulation of bacteria [4]. However, this balance can be compromised under certain conditions, leading to a state of ‘dysbiosis’ characterized by a reduction in defense mechanisms [5]. The loss of this balance can, in turn, progress to a state of ‘pathobiosis’ dependent on various factors (such as hormonal levels, sexual practices, lifestyle, bacterial interactions, and immune defenses), resulting in increased susceptibility to vaginitis and sexually transmitted diseases (STDs) [6, 7]. Therefore, a vaginal ecosystem in eubiosis represents a state of well-being for women as there is a lower susceptibility to vaginal infections [8]. At the vaginal level, more than 20 different species of Lactobacilli have been recognized [9]. However, most healthy women predominantly have a few species represented, including *L. crispatus, L. iners*, *L. jensenii*, and *L. gasseri*. In addition, other microorganisms such as Staphylococcus, Ureaplasma, Streptococcus, Gardnerella, Mycoplasma, Enterococcus, Escherichia coli, and Candida are present, albeit in smaller quantities [10]. Lactobacilli are involved in maintaining normal conditions within the vaginal ecosystem by preventing the uncontrolled growth of pathogenic and opportunistic microorganisms [11]. The main mechanisms through which lactobacilli exert their protective function are represented by competition with other microorganisms for nutrients and adherence to the vaginal epithelium, reduction of vaginal pH through the production of organic acids, especially lactic acid, production of antimicrobial substances, such as bacteriocins and hydrogen peroxide, and modulation of the local immune system [12]. However, the microbial communities present in the vagina can undergo variations in their representation due to factors such as age, sexual activity, pharmacological treatment, and hygiene [13]. Therefore, the homeostasis of the vaginal ecosystem is the result of complex interactions and synergies between the host and the various microorganisms that colonize the vaginal mucosa; maintaining a high number of resident lactobacilli is an effective indicator of women’s health [14]. An abnormal flora, characterized by a drastic reduction or disappearance of lactobacilli, underlies some vaginal pathologies such as bacterial vaginosis and aerobic vaginitis [15]. Existing literature discussing whether there is and what the correlation is between eubiosis, dysbiosis, pathobiosis, fungal vaginitis, bacterial vaginosis, and STDs is unclear, and data on the correlation between STDs and vaginal pathobiosis are contradictory.

Thus, this study aimed to analyze the association between specific alterations found in fresh bacterioscopic examinations (FBE) and the risk of certain infections in patients with positive vaginal swabs.

## Materials and methods

This is a retrospective, monocentric, epidemiological study conducted from January 2013 to December 2023.

Patients were selected meeting the following criteria: women not pregnant; age between 18 and 69 years; sexually active women; Eastern Cooperative Oncology Group (ECOG) performance status 0–1; patients who underwent FBE and vaginal swabs. Patients with urinary or genital tract infections or STDs under pharmacological treatment, pregnant or lactating patients, or women with other gynecological conditions causing bleeding (polyps, endometrial hyperplasia, etc.) were excluded.

For the FBE using phase contrast microscopy, a sample of vaginal discharge was collected from the posterior vaginal fornix and the lateral vaginal fornices. This sample was then placed on two slides, one containing a drop of saline solution (0.9% NaCl) and the other containing a drop of KOH solution. After placing the collected material on the second slide, the sniff test was performed to assess for the presence of a ‘fishy odor’ as suggested by the Amsel criteria for the diagnosis of bacterial vaginosis [16]. A portion of the discharge was brought into contact with litmus paper to analyze its pH. The two slides were observed under a phase contrast microscope at a magnification of first 200 × and then 400 × for an area of 0.016 mm^2^.

Based on observations obtained from the FBE, the patients were divided into two groups.

The control group consisted of women with a vaginal ecosystem in eubiosis; this group included patients who presented physiological pH and a vaginal ecosystem in eubiosis on FBE, meaning the absence of inflammation and the presence of rod-shaped bacterial forms (Döderlein’s bacillus); vaginal cells exhibited clear cytoplasm with slightly eccentric nuclei; vaginal swabs for Chlamydia, Mycoplasma, Gonococcus, common bacteria, and fungi, as well as human papillomavirus (HPV) PCR with segment analysis, were all negative; and negative fish odor test.

The pathological group included women with dysbiosis in the vaginal ecosystem; this group encompassed patients who exhibited various abnormalities during FBE, including alterations in vaginal pH or alkaline pH, presence of lactobacilli with inflammation (indicated by a higher count of leukocytes compared to shedding epithelial cells), presence of Döderlein’s cytolysis (characterized by crumpled cells due to excessive lactic acid produced by lactobacilli), presence of spores or hyphae suggestive of fungal vaginitis, presence of mixed bacterial flora (with an altered ratio of lactobacilli to cocci, with cocci flora prevailing), presence of Trichomonas vaginitis, presence of clue cells without leukocytes (indicative of bacterial vaginosis), and positive fish odor test. Within this group, patients were further categorized into five subgroups based on specific infections or the presence of co-infections. These subgroups were: a) positive vaginal swab for *Chlamydia trachomatis*; b) positive vaginal swab for Mycoplasma; c) positive vaginal swab for common bacteria; d) positive vaginal swab for HPV; and e) multiple positive vaginal swabs indicating co-infections. Patients who solely exhibited abnormalities in FBE without any of the specific infections analyzed were excluded from the study.

The statistical analysis was conducted using SPSS software (SPSS Inc., Chicago, IL, USA); a confidence interval (CI) of 95% was chosen, and a *p* value of 0.05% was considered statistically significant to test the hypothesis. For each group, the percentage relative to the total sample was calculated, as well as the median age of the patients included and the distribution of the data considering the standard deviation (SD). The quantitative variables present between the two study groups were compared using the Chi-square test with a *p* value set at 0.05%. Meanwhile, the comparison between repeated measures was conducted using the analysis of variance (ANOVA) test, with the values obtained through this test showing a normal distribution and equal variances of the considered variables. The results were reported as positive or negative deviations from SD. All tests used were two-tailed.

## Results

In total, 2110 patients were included, with ages ranging from 18 to 69 years. The median age was 33 ± 11.26 years.

Within the control group, 811 patients (38.4%) aged 18 to 69 years were included, accounting for 38% of the total sample analyzed. The median age was 32 ± 11.38 years.

Among the pathological group, there were 1299 patients (61.6%) aged between 18 and 69 years, constituting 62% of the overall sample. The median age was 33 ± 11.36 years. Among these patients, 114 had negative vaginal swabs (8.8% of positive FBE), while the remaining 1185 patients (91.2% of positive FBE) with positive vaginal swabs were distributed among 5 subgroups.

The subgroup related to women positive for *Chlamydia trachomatis* included 85 patients, which was 7% of the women with one or more infections associated with abnormalities on FBE. In particular, in two patients, this infection was associated with the presence of lactobacilli and inflammation on FBE, and Chlamydia was associated with 2% of this specific abnormality. In 31 patients, it was associated with the presence of mixed bacterial flora, accounting for 7% of this specific abnormality on FBE. In 40 patients, it was associated with the presence of bacterial vaginosis, representing 9% of all infections associated with this specific abnormality on FBE. In addition, two women, or 2% of the analyzed sample, showed the presence of Trichomonas on FBE, and finally, five patients in this group, 2% of the total, exhibited a concurrent fungal infection. None of the patients with *Chlamydia trachomatis* infection showed a picture of Döderlein’s cytolysis on FBE.

A total of 192 patients who tested positive for *Mycoplasma hominis* and Ureaplasma in vaginal swabs were included, constituting 16% of women with one or more infections detected during FBE. Notably, among these patients, 16 exhibited this infection alongside the presence of lactobacilli and inflammation on FBE, with Mycoplasma associated with 14% of this specific abnormality. In 77 patients, these infections were linked to mixed bacterial flora, accounting for 16% of this abnormality on FBE. Furthermore, in 99 patients, these infections were associated with bacterial vaginosis, representing 22% of all infections linked to this specific abnormality on FBE. In addition, none of the women showed evidence of Trichomonas, concurrent fungal infections, or Döderlein’s cytolysis during FBE.

Two hundred thirty-eight patients who tested positive for common bacteria in vaginal swabs were included, comprising 20% of women with one or more infections detected during FBE. Specifically, among these patients, 46 showed an association between this infection and the presence of lactobacilli and inflammation on FBE, with common bacteria accounting for 41% of this specific abnormality. In 34 patients, an association with the presence of fungi on FBE was observed, constituting 16% of all infections linked to this particular FBE abnormality. Moreover, in 94 patients, these bacteria were linked to mixed bacterial flora, representing 20% of this specific abnormality on FBE. In two women, they were even detected in the presence of *Trichomonas vaginalis* observed under the microscope, constituting 18% of all infections specifically associated with this type of observation. In addition, in 28 patients, they were linked to the presence of bacterial vaginosis, representing 6% of all infections associated with this specific abnormality on FBE. Notably, none of the patients with *Chlamydia trachomatis* infection exhibited signs of Döderlein’s cytolysis during FBE.

Two hundred eighty-eight patients who tested positive for HPV in vaginal swabs were included, comprising 24% of women with one or more infections detected during FBE. Among these patients, ten showed an association between this infection and the presence of lactobacilli and inflammation on FBE, with HPV accounting for 9% of this specific abnormality. In addition, in 33 women, an association with Döderlein’s cytolysis was observed, constituting 58% of all infections associated with this alteration. Furthermore, in 37 patients, an association with the presence of fungi on FBE was found, constituting 18% of all infections linked to this FBE abnormality. Moreover, in 83 patients, HPV was linked to mixed bacterial flora, representing 18% of this abnormality on FBE. In two women, it was even detected in the presence of *Trichomonas vaginalis* observed under the microscope, constituting 18% of all infections specifically associated with this type of observation. In addition, in 123 patients, it was associated with the presence of bacterial vaginosis, representing 28% of all infections associated with this specific abnormality on FBE.

Four hundred twenty-one patients with vaginal swabs positive for more than one infection or presence of co-infections were included, accounting for 36% of women with one or more infections associated with abnormalities on FBE. In particular, in 30 patients, these infections were associated with the presence of lactobacilli and inflammation on FBE, and co-infections were associated with 27% of this abnormality. In three women, an association with Döderlein’s cytolysis was detected, constituting 5% of all infections associated with this alteration. In 62 patients, an association with the presence of fungi on FBE was found, constituting 30% of all conditions correlated with this specific FBE abnormality. In 181 patients, it was associated with the presence of mixed bacterial flora, accounting for 38% of this abnormality on FBE. In three women, it was even detected in the presence of *Trichomonas vaginalis* observed under the microscope, constituting 27% of all infections specifically associated with this type of observation. In 142 patients, it was associated with the presence of bacterial vaginosis, representing 32% of all infections correlated with this specific abnormality on FBE.

The types of microorganisms affecting the patients of the five subgroups and the respective FBE alterations are shown in Table 1.

**Table 1:** Rates of fresh bacterioscopic examination alterations and microorganisms in patients with positive vaginal swabs

	Chlamydia (SD 4.66)	Mycoplasma (SD 40.87)	Common bacteria (SD 34.17)	HPV (SD 56.26)	Coinfections (SD 80.35)	No microrganisms (SD 23.54)	Total (SD 193.3)
Lactobacilli with inflammation (*n* = 111)	2 (2%) *P* = 0.671*	16 (14%) *P* = 0.662*	46 (41%) *P* = 0.006**	10 (9%) *P* = 0.763*	30 (27%) *P* = 0.106**	7 (6%)	111
Cytolysis (*n* = 56)	0 (0%) *P* = 0.999**	0 (0%) *P* = 0.999**	0 (0%) *P* = 0.999**	33 (58%) *P* = 0.278**	3 (5%) *P* = 0.999**	20 (36%)	56
Fungi (*n* = 208)	5 (2%) *P* = 0.767**	0 (0%) *P* = 0.480**	34 (16%) *P* = 0.217**	37 (18%) *P* = 0.743**	62 (30%) *P* = 0.201**	70 (34%)	208
Mixed bacterial flora (*n* = 470)	31 (7%) *P* = 0.479*	77 (16%) *P* = 0.229**	94 (20%) *P* = 0.001**	83 (18%) *P* = 0.095**	181 (38%) *P* = 0.174**	4 (0.8%)	470
*Trichomonas vaginalis* (*n* = 11)	2 (2%) *P* = 0.999**	0 (0%) *P* = 0.999**	2 (18%) *P* = 0.999**	2 (18%) *P* = 0.999**	3 (27%) *P* = 0.999**	2 (18%)	11
Vaginosis (*n* = 443)	40 (9%) *P* = 0.001**	99 (22%) *P* > 0.001**	28 (6%) *P* = 0.813**	123 (28%) *P* = 0.343**	142 (32%) *P* = 0.001**	11 (2%)	443
Total 1299	80 (6%)	192 (15%)	204 (16%)	288 (22%)	421 (32%)	114 (9%)	1299

*HPV* human papillomavirus; *SD* standard deviation, *Chi-square test; **Fisher test

Subsequently, an analysis of two subgroups of patients based on two specific alterations in the FBE was performed: women who presented mixed bacterial flora and those in whom the examination, together with other diagnostic criteria, suggested the diagnosis of bacterial vaginosis. After identifying the prevalence for each of these subgroups, the specific common germs that were most involved in the analyzed cases were determined.

All patients whose FBE suggested the presence of mixed bacterial flora were selected, i.e., all cases where the number of cocci observed was equal to or greater than the number of lactobacilli present. This sample consisted of 470 women (SD 59.92), and within this specific subgroup, the presence of patients with vaginal swabs positive for common germs was sought. Specifically, 87 women with this characteristic were identified (19% of all those who presented mixed bacterial flora upon microscopic observation). At this point, the types of common germs that were most involved in these infections were analyzed to determine their frequency and their association with this specific alteration observed in the FBE. It emerged that the most associated common germs with this form of dysbiosis were *Escherichia coli* and Enterococcus, both present in 7% of the cases examined (in 31 and 30 patients, respectively), followed by Streptococcus, present in 6% of the cases (26 women), and others (Klebsiella, Proteus, etc.) present in 3% of the cases (13 women). These bacteria were identified through the execution of vaginal swabs.

All individuals whose recent bacterioscopic analysis indicated potential vaginosis were chosen, encompassing instances where clue cells were detected without signs of inflammation, accompanied by or without a fishy odor during a sniff test conducted using a KOH solution. This cohort comprised 443 women (SD 63.98). Within this specific subset, efforts were made to identify patients whose vaginal swabs tested positive for common germs. Precisely, 43 women exhibiting this trait were recognized (constituting 10% of all positively diagnosed cases of vaginosis). Subsequently, an exploration was conducted to ascertain which types of common germs predominantly contributed to these infections, delineating their prevalence and correlation with the aforementioned alteration noted during the FBE. It was revealed that Streptococcus was the most frequently implicated common germ in this dysbiosis manifestation, accounting for 3% of the analyzed cases (12 patients), followed by Enterococcus at 2% (11 patients). In addition, *Escherichia coli* was detected in 2% of patients (ten women), alongside other common germs such as Klebsiella and Proteus, each found in 2% of cases among the ten patients examined. These bacteria were identified through the collection and analysis of vaginal swabs.

## Discussion

The present analysis demonstrated the correlation between the dysbiosis state and the increased predisposition to vulvovaginal infections and major STDs.

First, it was observed that the pathological group consisted of a larger number of patients who attended the outpatient clinics. Within this group of women, characterized by vaginal microbiota dysbiosis observed through FBE, there was also a higher prevalence of vaginal infections detected through vaginal swabs.

In particular, the alteration detected in FBE with the highest frequency was the presence of mixed bacterial flora correlated with a greater prevalence of co-infections. That is to say, in the presence of mixed bacterial flora and a marked reduction of lactobacilli, it was more likely that different pathogens simultaneously predominate, further worsening the imbalance and causing significant symptomatology. A similar prevalence, however, was observed for common germs and infections from various strains of HPV.

The second most common alteration detected in FBE was bacterial vaginosis, also highly associated with the presence of co-infections and HPV, and with a lower prevalence of common germs, Mycoplasma, and Chlamydia. These results are consistent with a study by Balkus et al. [17], in which a similar prevalence of vaginal infections and pathogens was observed in patients with bacterial vaginosis. In this study, 221 women underwent monthly vaginal swabs, which showed prevalence of bacterial STDs (*Chlamydia trachomatis*, *Neisseria gonorrhoeae*, or *Mycoplasma genitalium* infection) very similar to those recorded in the present study.

The third alteration detected in FBE in terms of frequency was the presence of fungi, also strongly associated with the presence of co-infections and HPV, in accordance with the results obtained by Boselli et al. [18], including 1644 women, in which a presumed diagnosis of vulvovaginal infection was determined based on specific clinical and laboratory criteria (including pH and sniff tests) in 902 cases (55.4%), while a definitive diagnosis was established in 1439 cases (87.5%); the definitive diagnoses were categorized as follows: vulvovaginal mycosis in 844 cases (51.3%), bacterial vaginosis in 327 cases (19.9%), Trichomonas infection in 110 cases (6.7%), aspecific bacterial vaginitis in 100 cases (6.1%), and non-infectious vaginitis in 58 cases (3.5%). *Candida albicans* infections accounted for the majority (78%) of mycosis cases as determined by typing, totaling 459 cases [18].

Finally, the alteration observed with the lowest frequency was Döderlein’s cytolysis. The latter, also known as lactobacillus cytolysis, refers to the breakdown or lysis of lactobacilli, which are beneficial bacteria found in the vaginal microbiota [2]. These bacteria play a crucial role in maintaining vaginal health by producing lactic acid, which helps to maintain an acidic pH and prevent the overgrowth of harmful bacteria or pathogens [3]. When Döderlein’s cytolysis occurs, there is a disruption in the balance of the vaginal microbiota, leading to the destruction of lactobacilli [2]. This can result in a decrease in vaginal acidity, making the environment more conducive to the growth of other bacteria or fungi, potentially leading to vaginal infections or discomfort [3]. A retrospective analysis conducted in Brazil [2] examined the presence of various microorganisms and cytological abnormalities in vaginal, cervical, and endocervical samples collected from healthy women of reproductive age between 1994 and 2004, totaling approximately 14,000 participants. The paper focused on identifying *Candida albicans*, *Trichomonas vaginalis*, clue cells, Döderlein’s bacilli, cytolytic flora, coccoid bacillus, cervical intraepithelial neoplasia (CIN), and HPV. The cytological samples were categorized into proliferative and secretory phases. Results revealed that the frequency of cytolytic flora and *Candida albicans* varied depending on the phase of the menstrual cycle, while menstrual cycle phases did not influence CIN.

From the present study, a strong correlation emerged between the presence of specific alterations in the FBE and an increased prevalence of certain infections. Specifically, the concurrent presence of lactobacilli and typical inflammatory cells was associated with a higher frequency of positive swabs for common germs, which often lead to co-infections. Conversely, when Döderlein’s cytolysis was observed in the FBE, there was a clear predominance of positive swabs for HPV. Therefore, in patients exhibiting this specific alteration in the FBE, further investigation with molecular swabs for HPV detection would be advisable. In cases where spores and fungal hyphae were present in the FBE, there was a higher prevalence of co-infections, suggesting that vulvovaginitis in many cases is not solely of fungal origin. A similar consideration can be made when mixed bacterial flora is observed under the microscope. In cases where *Trichomonas vaginalis* was detected in the FBE, there was a similar likelihood of positive swabs for other pathogens, except for Mycoplasma, which seems to be less prevalent in these cases. Finally, bacterial vaginosis was more frequently associated with the presence of co-infections and HPV infections, thus specific swabs for the detection of these germs are suggested in these cases. From these observations, the importance of performing an FBE in all patients exhibiting signs and symptoms of vulvovaginal infections emerges that the prevalence of positive vaginal swabs for the most common pathogens was high (57%) as it is an easy-to-perform and cost-effective technique. Furthermore, it allows to decide whether to proceed with vaginal swabs as a second-level diagnostic technique and, potentially, which ones might be most important based on microscopic observations. In addition, it could have therapeutic implications by enabling to prompt administration of targeted therapy [4]. An important role could also be played by concurrent therapy with probiotics [5] because they would help restore the eubiotic state of the vulvovaginal bacterial flora, which appears to be a protective factor [6].

One of the notable strengths of this study is the ability to compare results from FBE and vaginal swabs conducted within the same facility, thereby minimizing biases associated with laboratory analyses. Nonetheless, this analysis is not without its limitations. One primary concern is the relatively smaller size of the control group when compared to the pathological group. In addition, as this research was conducted at a single center, it faces the significant constraint of not being generalizable to the broader population. Another limitation is the lack of data on the long-term outcomes following the various pharmacological treatments provided. Consequently, to solidify these findings, it is imperative to undertake further prospective, multi-centric studies with more extended follow-up periods.

## Conclusion

The state of vaginal balance plays a crucial role in predisposing women to major infectious pathologies of the vagina and vulva. Specifically, a state of eubiosis, characterized by a clear predominance of lactobacillus species over other microorganisms normally present in the vaginal bacterial flora, appears to be protective against STDs, vaginitis, and bacterial vaginosis. Similarly, an imbalance in the vaginal bacterial flora, with reduced lactobacilli, predisposes to a greater susceptibility to major vulvovaginal infections and STDs. A strong correlation emerges between the presence of specific alterations in the FBE and an increased prevalence of certain infections. These findings may potentially influence clinical management and therapeutic approaches in these patients.

## Data Availability

The data that support the findings of this study are available from the corresponding author, upon reasonable request.
